# First Evidence of Immunomodulation in Bivalves under Seawater Acidification and Increased Temperature

**DOI:** 10.1371/journal.pone.0033820

**Published:** 2012-03-27

**Authors:** Valerio Matozzo, Andrea Chinellato, Marco Munari, Livio Finos, Monica Bressan, Maria Gabriella Marin

**Affiliations:** 1 Department of Biology, University of Padova, Padova, Italy; 2 Department of Statistical Sciences, University of Padova, Padova, Italy; MESC - University of South Alabama, United States of America

## Abstract

Water acidification, temperature increases and changes in seawater salinity are predicted to occur in the near future. In such a global climate change (GCC) scenario, there is growing concern for the health status of both wild and farmed organisms. Bivalve molluscs, an important component of coastal marine ecosystems, are at risk. At the immunological level, the ability of an organism to maintain its immunosurveillance unaltered under adverse environmental conditions may enhance its survival capability. To our knowledge, only a few studies have investigated the effects of changing environmental parameters (as predicted in a GCC scenario) on the immune responses of bivalves. In the present study, the effects of both decreased pH values and increased temperature on the important immune parameters of two bivalve species were evaluated for the first time. The clam *Chamelea gallina* and the mussel *Mytilus galloprovincialis*, widespread along the coast of the Northwestern Adriatic Sea, were chosen as model organisms. Bivalves were exposed for 7 days to three pH values (8.1, 7.7 and 7.4) at two temperatures (22 and 28°C). Three independent experiments were carried out at salinities of 28, 34 and 40 PSU. The total haemocyte count, Neutral Red uptake, haemolymph lysozyme activity and total protein levels were measured. The results obtained demonstrated that tested experimental conditions affected significantly most of the immune parameters measured in bivalves, even if the variation pattern of haemocyte responses was not always linear. Between the two species, *C. gallina* appeared more vulnerable to changing pH and temperature than *M. galloprovincialis*. Overall, this study demonstrated that climate changes can strongly affect haemocyte functionality in bivalves. However, further studies are needed to clarify better the mechanisms of action of changing environmental parameters, both individually and in combination, on bivalve haemocytes.

## Introduction

Global climate changes (GCCs) are occurring presently and are predicted to continue in the coming decades through changes in abiotic environmental factors, such as temperature increases, water acidification, and changes in precipitation and seawater salinity [1]. Among the processes responsible for GCCs, the increasing level of carbon dioxide (CO_2_) in the atmosphere is considered to be one of the most important. Furthermore, high levels of atmospheric CO_2_ are expected to affect average air and ocean temperatures, to cause widespread melting of snow and ice and to raise the average sea level [1]. Pre-industrial atmospheric CO_2_ levels were approximately 280 ppm, whereas at present, they have increased to more than 380 ppm, mainly due to anthropogenic activities [2]. The capability of the oceans to take up CO_2_ may influence seawater carbonate chemistry, with a consequent decrease in pH values, concentration of carbonate ions and the related calcium carbonate (CaCO_3_) saturation state of seawater [3]. Several reports have implied that the continued release of CO_2_ into the atmosphere has already caused a reduction in ocean pH values of approximately 0.1 pH units with respect to the pre-industrial levels and that reductions from 0.3 to 0.5 pH units are predicted to occur before the end of the 21^st^ century [1], [4]–[6]. Increasing atmospheric CO_2_ levels may cause a pH reduction of 0.7 units by 2300 [7].

Studies have also reported that the mean global temperature has increased by approximately 0.7°C during the last century and that further increases are expected during the present century [1], [8]. The global temperature is predicted to increase by approximately 1.8 to 4.0°C by the end of the 21^st^ century [1]. However, warming is expected to occur heterogeneously, with land warming faster than the oceans, high latitudes warming faster than the mid-latitudes, and winters warming more than summers [1].

Alterations in seawater salinity values, mainly in estuarine and coastal areas, give cause for concern [9], [10]. Global warming should be associated with changes in the hydrological cycle at a large scale: increases in precipitation could occur at high latitudes and near the tropics, whereas decreases could occur in the sub-tropical and mid-latitude regions [11]; therefore, many areas will experience increasing drought or flooding. Frequent flood events are hypothesised to lead to prolonged and more frequent periods of reduced salinity, particularly in estuarine areas [12].

In a GCC scenario, increasing concern is being directed to the health status of living organisms because GCCs could affect population dynamics and the distribution of livestock parasites. Increases in temperature are expected to have marked effects on the abundance of parasite populations, in particular, due to higher rates of development and releasing of infective stages [13], [14], and could change the geographic distributions of many parasites [15], [16]. As a consequence, increases in disease occurrence and production loss could occur [17], [18]. In this context, it is crucial to understand if and how living organisms cope with these changing stress conditions, considering that defence capabilities differ among species. At the immunological level, responses comprise a complex network of specific and non-specific humoral and cell-mediated components. In invertebrates, such as bivalve molluscs, the immune system is relatively simple and lacks antibody-mediated specificity and memory. As a consequence, invertebrate immune functions are not adaptive but innate [19]. In bivalve cell-mediated immune responses, the main defence against pathogens and foreign materials is phagocytosis by circulating haemocytes; however, important roles are played by hydrolytic and oxidative enzymes, reactive oxygen species (ROS), reactive nitrogen intermediates, and antimicrobial peptides [19]–[21].

Despite detailed data available in the literature about the involvement of haemocytes in immune responses, limited information demonstrating the direct effects of GCC on bivalve immune parameters can be found [22]. To address this disparity, the present study evaluated for the first time the combined effects of decreased pH values and high temperatures on important immune parameters of two bivalve species widespread along the coast of the North-western Adriatic Sea: the clam *Chamelea gallina* and the mussel *Mytilus galloprovincialis*. The two species were chosen as model organisms considering that marine coastal zones should be particularly vulnerable to climate changes and taking into account the important ecological and commercial role of bivalves. Both species are filter-feeders; *C. gallina* lives in the sandy bottom, whereas *M. galloprovincialis* is common on intertidal natural and artificial hard substrates and is cultured in many coastal areas. After 7 days of bivalve exposure to reduced seawater pH and high temperatures at differing salinities, total haemocyte count (THC), capability of haemocytes to take up the vital dye Neutral Red (NR), lysozyme-like activity in cell-free haemolymph (CFH), and CFH total protein levels were chosen as immunomarkers of climate changes.

## Materials and Methods

### Animals and the experimental plan

No specific permits were required for the described study. According to the guidelines of the ethic board C.E.A.S.A. (*Comitato Etico di Ateneo per la Sperimentazione Animale*) of the University of Padova, no specific permission is necessary for the studies involving invertebrates. In any case, we declare that the present study did not involve endangered or protected species and that stress was minimised during both animal samplings and experiments.

Specimens of *C. gallina* (2.5±0.2 cm shell length) and *M. galloprovincialis* (4±0.5 cm shell length) were collected along the west coast of the Northern Adriatic Sea (near Chioggia, Italy) and immediately transferred to the laboratory. Bivalves were carefully checked for shell damage (damaged animals were not used for experiments), and epibionts (such as barnacles and algae) were removed from the mussels. Before the experiments, bivalves were maintained in large aquaria with aerated seawater at salinity, temperature and pH values recorded in the field during animal sampling and were fed with microalgae (*Isochrysis galbana*). The experiments were performed far from periods of sexual maturity of mussels (i.e., in summer) and clams (i.e., in winter/spring); this not-reproductive condition avoids spawning and reduces possible additional stress during the experiments.

**Figure 1:**
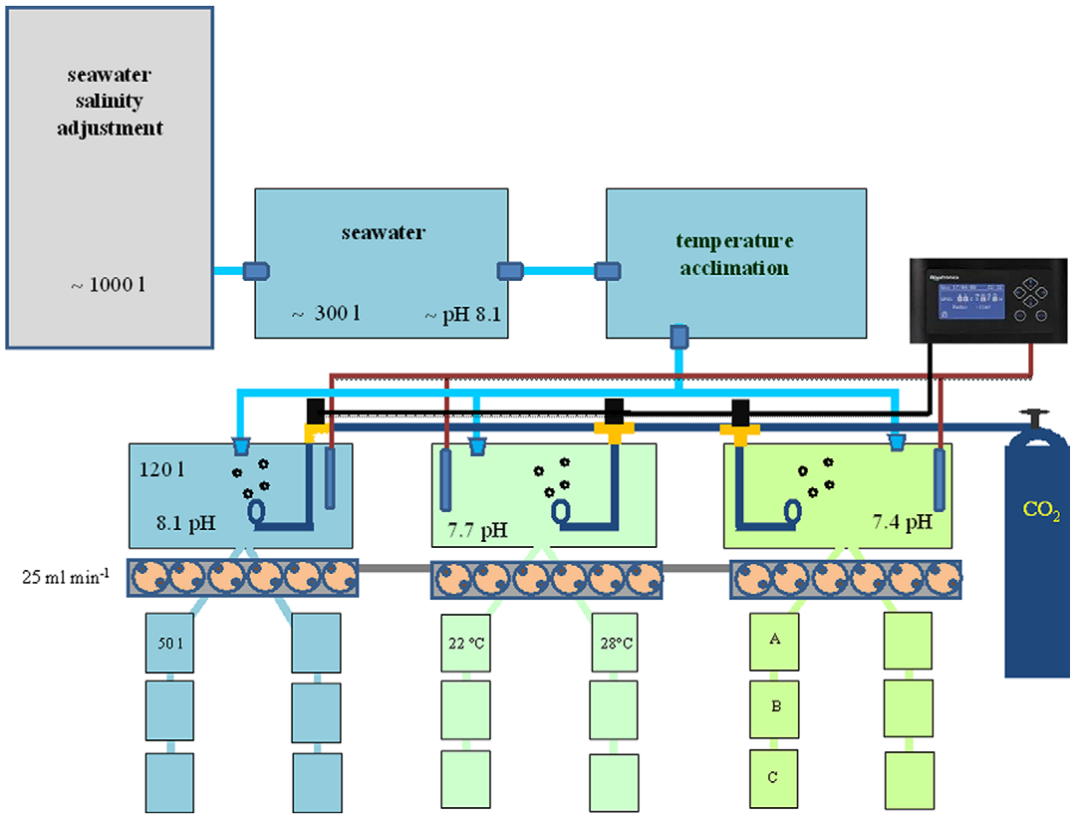
The experimental flow-through system. See M&M section for details.

The experimental flow-through system is depicted in Figure 1. The system consisted of a main reservoir filled with natural seawater (approximately 1000 l), where salinity was adjusted to experimental values (28, 34 and 40 PSU) by the addition of distilled water or artificial hypersaline seawater (*Red Sea Salt*, Red Sea Fish Pharm, Israel). The seawater was next pumped into two tanks (approximately 300 l each) used to allow suspended matter to settle and seawater to equilibrate to the controlled laboratory temperature. The seawater next moved on to three tanks of approximately 120 l capacity, where the pH was adjusted to experimental values by bubbling CO_2_ using an automatic control system (*Aquarium Controller Evolution*, mod. ACQ110, Aquatronica, Italy) connected with pH electrodes (ACQ310N-PH, Aquatronica, Italy). The seawater was finally pumped (25 ml min^−1^) into experimental tanks (A, B and C, approximately 50 l each) containing bivalves. Prior to starting exposure, bivalves were acclimatised to experimental conditions by gradually increasing or decreasing the seawater temperature (2°C day^−1^) to 22 and 28°C and by gradually decreasing pH values to 7.7 and 7.4. The water temperatures in the experimental tanks were maintained at constant values using electronic thermostats. Bivalves were exposed for 7 days to six combinations of pH and temperature (3 pH values at 2 different temperatures). Of the resulting experimental conditions, the combination of a pH of 8.1 and a temperature of 22°C was considered as a reference. For each species, three independent experiments were performed at the salinities of 28, 34 and 40 PSU. For each experimental condition, three replicate tanks were prepared (20 individuals per replicate). Bivalve mortality was checked daily. At the end of each experiment, haemolymph was collected to measure immune parameters.

### Haemolymph collection

Fifteen pools of haemolymph from 3 bivalves were prepared (i.e., 5 pools per replicate tank) for each set of experimental conditions. Haemolymph (approximately 300 µl per animal) was collected from the anterior adductor muscle with a 1-ml plastic syringe and stored in ice. An equal volume of 0.38% sodium citrate (Sigma) in 0.45 µm-filtered sea water (FSW) with a pH of 7.5 was added to haemolymph samples to prevent clotting. One hundred microlitres of pooled haemolymph were used to determine the THC, and 500 µl of haemolymph was used to assay the NR uptake. One hundred microlitres of pooled haemolymph (without sodium citrate) was used to measure both lysozyme-like activity and total protein concentration in CFH.

### THC determination

A Model Z2 Coulter Counter electronic particle counter/size analyser (Coulter Corporation, FL, U.S.A.) was used to determine the THC. Pooled haemolymph (100 µl) was added to 19.9 ml of FSW. THC results were expressed as the number of haemocytes (×10^6^ or ×10^5^) ml haemolymph^−1^.

### Neutral Red (NR) uptake assay

NR uptake assay was performed according to the procedure reported in previous studies [23], [24]. Pooled haemolymph was centrifuged at 780×*g* for 10 min. Haemocytes (at a final concentration of 10^6^ cells ml^−1^) were resuspended in an equal volume of 8 mg l^−1^ NR dye (Merck) solution in FSW, and incubated at room temperature for 30 min. They were next centrifuged at 780×*g* for 10 min, re-suspended in distilled water, sonicated at 0°C for 30 s with a Braun Labsonic U sonifier at 50% duty cycles, and centrifuged at 12,000×*g* for 15 min at 4°C. The supernatant, corresponding to haemocyte lysate (HL) was collected for the NR uptake assay. Absorbance at 550 nm was recorded on a Beckman 730 spectrophotometer. The results were expressed as optical density per ml haemolymph (OD ml haemolymph^−1^).

### Haemolymph lysozyme activity assay

Lysozyme activity was quantified in CFH as an indicator of lysosomal membrane stability. Pooled haemolymph was centrifuged at 780 *g* for 10 min. The supernatant, corresponding to CFH, was collected, frozen and stored at −80°C before analyses. Fifty µl of CFH were added to 950 µl of a 0.15% suspension of *Micrococcus lysodeikticus* (Sigma) in a 66 mM phosphate buffer, with a pH of 6.2, and the decrease in absorbance (ΔA min^−1^) was continuously recorded at 450 nm for 5 min at room temperature. The results were expressed as µg lysozyme mg protein^−1^.

### Haemolymph protein concentration

Protein concentrations in CFH were quantified using bovine serum albumin as a standard [25]; a commercial kit (Quick Start Bradford protein assay, BIO-RAD, Hercules, CA, U.S.A.) was used. Twenty microlitres of CFH were incubated at room temperature for at least 5 minutes with 1 ml of 1× dye reagent. The absorbance was measured at 595 nm, and the results were expressed as mg protein ml haemolymph^−1^.

### Statistical analysis

Data were checked for normal distribution (Shapiro-Wilk test) and homogeneity of variances (Bartlett's test). As ANOVA assumptions were not fulfilled, a non-parametric approach was used for comparison of the results. For each salinity, the Permutational Analysis of Variance (PERMANOVA) with 999 permutations was performed to highlight significant effects of temperature, pH and temperature/pH interaction on the immunomarker responses. This statistical approach was chosen considering that three independent experiments were performed at three salinity values and that a preliminary overall PERMANOVA analysis on the entire dataset revealed significant effects of salinity on immunomarkers. In addition, the non-parametric Kruskal-Wallis test was followed by a *post hoc* test (with a Bonferroni correction for 5 comparisons) used to compare results obtained in bivalves from the reference condition (8.1 pH and 22°C) and those from the other experimental conditions. All results were expressed as the mean ± standard error (SE). The software packages STATISTICA 10 (StatSoft, Tulsa, OK, U.S.A.) and PRIMER 6 PERMANOVA Plus (PRIMER-E Ltd, Plymouth, UK) were used for the statistical analyses.

## Results

### *C. gallina*

**Figure 2:**
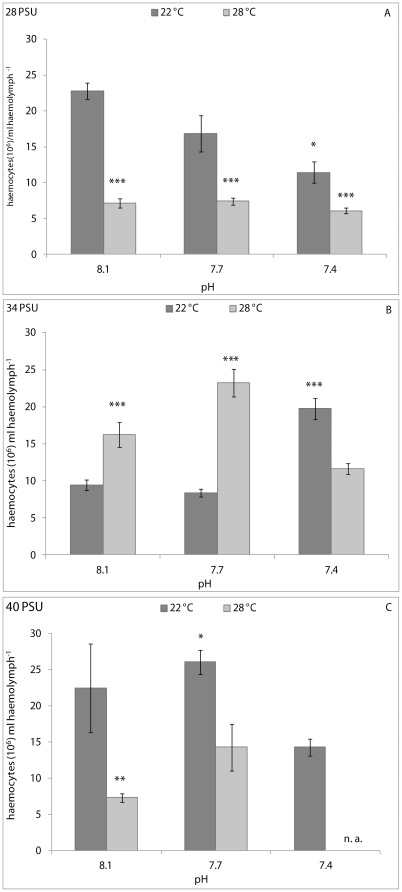
THC, expressed as number of haemocytes (10^6^) ml haemolymph^−1^, in *C. gallina* kept at 28 PSU (A), 34 PSU (B) and 40 PSU (C) salinities. Values are means ± SE; n = 15. Asterisks denote significant differences with respect to reference values (8.1 pH and 22°C temperature): *p<0.05, **p<0.01, ***p<0.001; n. a.: not available.

The PERMANOVA analysis results for the clams are showed in Table 1. Generally, significant effects of pH, temperature and their interaction on the immune parameters of clams were recorded. In particular, THC was significantly affected by pH (p<0.01), temperature (p<0.001) and their interaction (p<0.05) in clams kept at 28 PSU; by temperature (p<0.05) and pH/temperature interaction (p<0.001) at 34 PSU; and by pH (p<0.01) and temperature (p<0.05) at 40 PSU. At 28 PSU, pair-wise comparisons revealed a significantly higher THC in clams kept at 8.1 pH and 22°C, with respect to animals kept at the other experimental conditions, except for animals held at 7.7 pH and 22°C (Figure 2A). At a salinity of 34 PSU, significantly lower THC values were found in clams kept at 8.1 pH and 22°C, with respect to those of animals kept at 8.1 pH and 28°C (p<0.001); 7.7 and 28°C (p<0.001); and 7.4 pH and 22°C (p<0.001) (Figure 2B). At 40 PSU, significant differences in THC values were recorded between clams kept at 8.1 pH and 22°C, those held at 8.1 pH and 28°C (p<0.01) and those kept at 7.7 pH and 22°C (p<0.05) (Figure 2C).

**Table 1:** PERMANOVA analysis results.

*C. gallina*	source of variation	28 PSUp values	34 PSUp values	40 PSUp values
	temperature	**0.0010**	**0.0130**	**0.0020**
THC	pH	**0.0030**	0.2940	**0.0250**
	temperature X pH	**0.0130**	**0.0020**	0.6680
	temperature	**0.0030**	**0.0130**	**0.0010**
NRU	pH	**0.0010**	**0.0010**	**0.0030**
	temperature X pH	**0.0010**	**0.0020**	0.7440
	temperature	0.9130	0.4530	0.1730
LYS	pH	**0.0010**	0.7770	0.3370
	temperature X pH	**0.0010**	**0.0030**	0.2880
	temperature	**0.0010**	0.4230	0.1200
PROT	pH	**0.0010**	**0.0500**	0.0710
	temperature X pH	**0.0100**	**0.0060**	0.4790
***M. galloprovincialis***				
	temperature	**0.0270**	0.4690	**0.0010**
THC	pH	**0.0080**	**0.0030**	0.4440
	temperature X pH	0.6140	**0.0010**	**0.0150**
	temperature	0.1540	0.0910	0.2700
NRU	pH	0.0810	**0.0040**	**0.0110**
	temperature X pH	**0.0500**	**0.0210**	**0.0050**
	temperature	**0.0500**	0.1340	0.0950
LYS	pH	**0.0210**	0.8040	**0.0500**
	temperature X pH	0.2940	0.3240	**0.0110**
	temperature	**0.0430**	**0.0500**	0.2420
PROT	pH	**0.0010**	0.3710	**0.0010**
	temperature X pH	0.6360	0.2550	**0.0010**

For each immune parameter, statistically significant effects of the variables “temperature”, “pH” and “temperature X pH” interaction are indicated in bold. **THC**: total haemocyte count; **NRU**: neutral red uptake; **LYS**: haemolymph lysozyme activity; **PROT**: haemolymph protein concentration.

**Figure 3:**
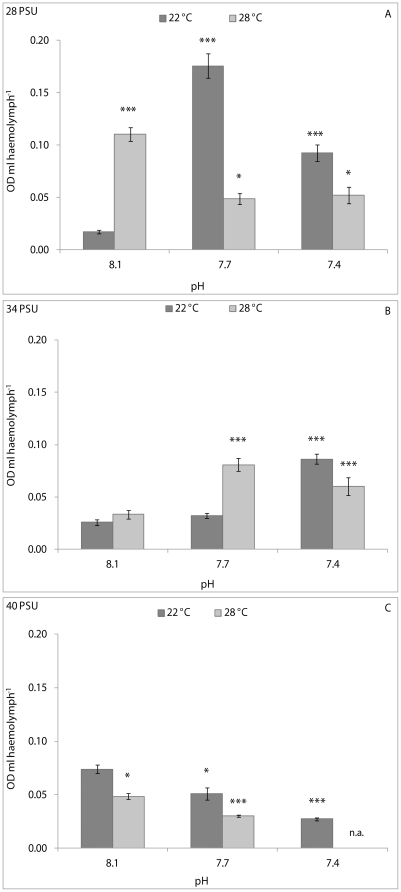
Neutral Red uptake, expressed as OD ml haemolymph^−1^, in *C. gallina* kept at 28 PSU (A), 34 PSU (B) and 40 PSU (C) salinities. Values are means ± SE; n = 15. Asterisks denote significant differences with respect to reference values (8.1 pH and 22°C temperature): *p<0.05, ***p<0.001; n. a.: not available.

NR uptake was affected significantly by pH, temperature and their interaction in clams kept at 28 and 34 PSU; and by pH and temperature at 40 PSU (see Table 1 for significance levels). At 28 and 34 PSU, pair-wise comparisons demonstrated that NR uptake by haemocytes from clams kept at 8.1 pH and 22°C was significantly lower than animals from the other experimental conditions, except for clams held at 7.7 pH and 22°C and at 8.1 pH and 28°C, both at 34 PSU (Figures 3A, 3B). An opposite pattern of variation in NR uptake was observed at 40 PSU, with dye levels being significantly higher in animals kept at 8.1 pH and 22°C compared with animals from the other experimental conditions (Figure 3C).

**Figure 4:**
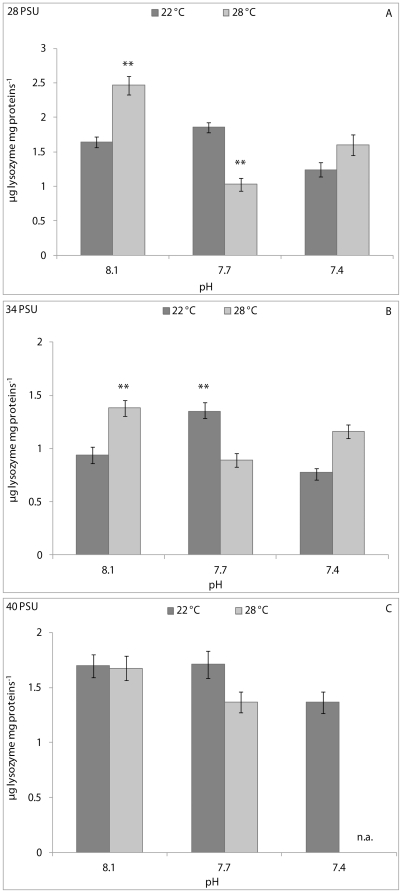
Lysozyme activity, expressed as µg lysozyme mg protein^−1^in cell-free haemolymph of *C. gallina* kept at 28 PSU (A), 34 PSU (B) and 40 PSU (C) salinities. Values are means ± SE; n = 15. Asterisks denote significant differences with respect to reference values (8.1 pH and 22°C temperature): **p<0.01; n. a.: not available.

Haemolymph lysozyme activity results were influenced significantly by pH and pH/temperature interaction (p<0.001) in clams kept at 28 PSU, and only by pH/temperature interaction (p<0.01) at 34 PSU (Table 1). At a salinity of 28 PSU, the CFH lysozyme activity of bivalves kept at 8.1 pH and 22°C was significantly (p<0.01) lower than that of animals kept at 8.1 pH and 28°C, and significantly higher than that of animals kept at 7.7 pH and 28°C (Figure 4A). At 34 PSU, the CFH lysozyme activity of clams kept at 8.1 pH and 22°C was significantly (p<0.01) lower than that of animals kept at 8.1 pH and 28°C, and at 7.7 pH and 22°C (Figure 4B). No significant differences in CFH lysozyme activity were observed at 40 PSU (Figure 4C).

PERMANOVA analysis revealed significant effects of pH (p<0.001), temperature (p<0.001) and their interaction (p<0.01) on CFH protein concentrations in clams kept at 28 PSU, and of pH (p<0.05) and pH/temperature interaction (p<0.01) at 40 PSU. At 28 PSU, CFH total protein levels of clams kept at 8.1 pH and 22°C were significantly (p<0.001) lower than those of animals held at 8.1 and 7.7 pH and 28°C, however, they were significantly (p<0.001) higher with respect to animals kept at 7.4 pH at both of the experimental temperatures (Table 2). At 34 PSU, CFH total protein levels of clams kept at 8.1 pH and 22°C were significantly lower than those of animals held at 7.7 pH and 28°C (p<0.01) and animals held at 7.4 pH and 22°C (p<0.001) (Table 2). No significant variations in the CFH total protein concentrations were observed at 40 PSU (Table 2).

**Table 2:** Total protein concentrations (mg ml^−1^) in haemolymph from *C. gallina*.

*C. gallina*			
experimental conditions	28 PSU	34 PSU	40 PSU
**8.1 pH, 22°C**	0.97±0.16	1.13±0.10	0.87±0.10
**8.1 pH, 28°C**	1.52±0.08***	1.08±0.07	0.75±0.11**
**7.7 pH, 22°C**	0.81±0.08	1.10±0.08	0.95±0.12
**7.7 pH, 28°C**	1.12±0.07***	1.36±0.16**	0.88±0.03
**7.4 pH, 22°C**	0.80±0.09***	1.66±0.13***	0.75±0.04**
**7.4 pH, 28°C**	0.75±0.10***	1.13±0.13	n.a.

Asterisks denote significant differences with respect to reference values (8.1 pH and 22°C temperature):

** p<0.01,

*** p<0.001; n. a.: not available. Results are means ± SE.

### *M. galloprovincialis*

**Figure 5:**
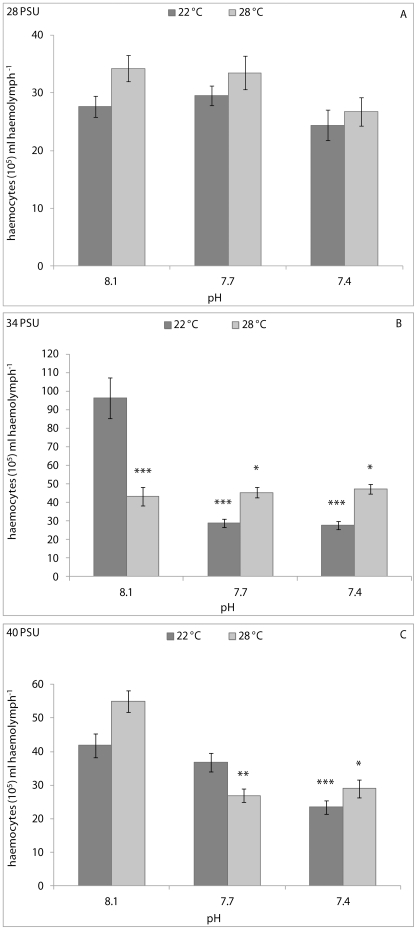
THC, expressed as number of haemocytes (10^5^) ml haemolymph^−1^, in *M. galloprovincialis* kept at 28 PSU (A), 34 PSU (B) and 40 PSU (C) salinities. Values are means ± SE; n = 15. Asterisks denote significant differences with respect to reference values (8.1 pH and 22°C temperature): *p<0.05, **p<0.01, ***p<0.001; n. a.: not available.

The PERMANOVA analysis results for mussels are reported in Table 1. Statistically significant effects of pH (p<0.001) and temperature (p<0.05) on the THC were observed in mussels kept at 28 PSU. A significant effect of pH (p<0.01) and pH/interaction (p<0.001) on THC were found in mussels kept at 34 PSU, whereas temperature (p<0.001) and pH/temperature interaction (p<0.05) affected the THC in mussels kept at 40 PSU. At a salinity of 28 PSU, pair-wise comparisons did not display any significant variation in THC between animals from the reference condition (8.1 pH and 22°C) and the others (Figure 5A), whereas at 34 PSU, reference mussels demonstrated significantly higher THC values with respect to animals kept at the other experimental conditions (Figure 5B). At 40 PSU, the THC of clams kept at 8.1 pH and 22°C was significantly higher than animals held at 7.7 pH and 28°C (p<0.01), and 7.4 pH at both the experimental temperatures (p<0.001 at 22°C and p<0.05 at 28°C) (Figure 5C).

**Figure 6:**
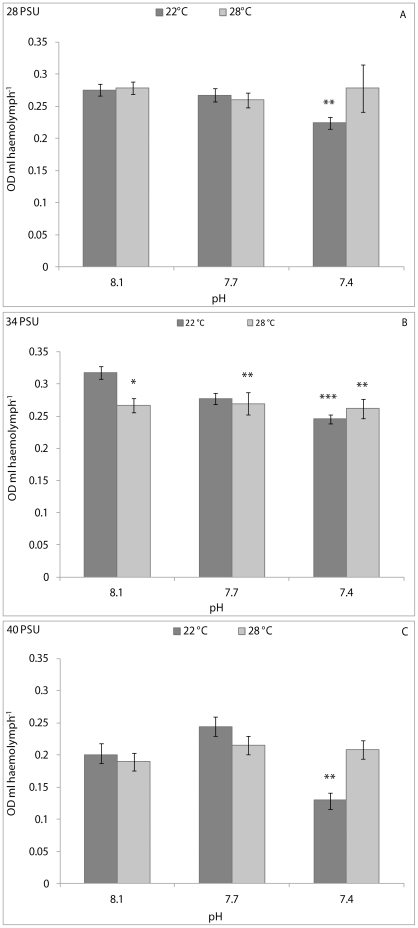
Neutral Red uptake, expressed as OD ml haemolymph^−1^, in *M. galloprovincialis* kept at 28 PSU (A), 34 PSU (B) and 40 PSU (C) salinities. Values are means ± SE; n = 15. Asterisks denote significant differences with respect to reference values (8.1 pH and 22°C temperature): *p<0.05, ***p<0.001; n. a.: not available.

The NR uptake was influenced significantly (p<0.05) by pH/temperature interaction in mussels kept at 28 PSU, and by pH and pH/temperature interaction at 34 PSU (p<0.01 and p<0.05, respectively) and at 40 PSU (p<0.05 and p<0.01, respectively) (Table 1). The pair-wise comparison demonstrated that mussels kept at 8.1 pH, 22°C and 28 PSU had significantly (p<0.01) higher NR uptake with respect to animals held at 7.4 pH and 22°C (Figure 6A). At a salinity of 34 PSU, a significantly higher NR uptake was observed in mussels kept at 8.1 pH and 22°C compared with that from the other experimental conditions except for 7.7 pH and 22°C (Figure 6B). At a salinity of 40 PSU, a significantly (p<0.01) increased NR uptake was found in clams kept at 8.1 pH and 22°C with respect to those at 7.4 pH and 22°C (Figure 6C).

**Figure 7:**
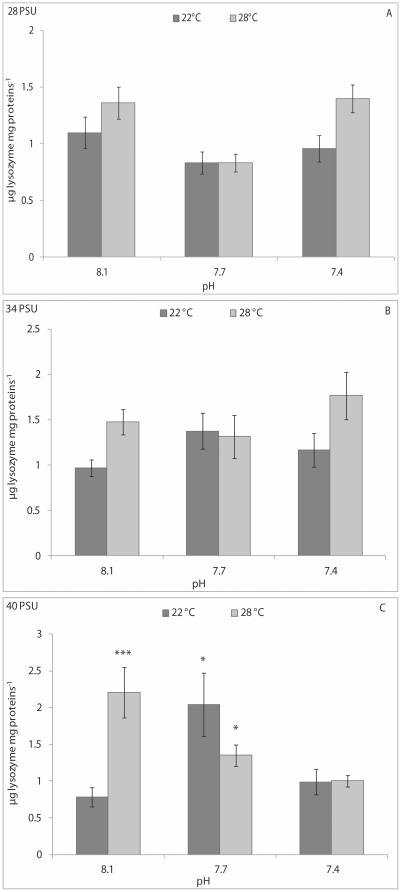
Lysozyme activity, expressed as µg lysozyme mg protein^−1^ in cell-free haemolymph of *M. galloprovincialis* kept at 28 PSU (A), 34 PSU (B) and 40 PSU (C) salinities. Values are means ± SE; n = 15. Asterisks denote significant differences with respect to reference values (8.1 pH and 22°C temperature): **p<0.01; n. a.: not available.

PERMANOVA analysis revealed a statistically significant effect of pH (p<0.05) and temperature (p<0.05) on the CFH lysozyme activity of mussels kept at 28 PSU. Significant effects of pH (p<0.05) and pH/temperature interaction (p<0.05) on enzyme activity were also found at 40 PSU. At salinities of 28 and 34 PSU, pair-wise comparisons did not highlight significant changes in the CFH lysozyme activity between animals from the reference condition (8.1 pH and 22°C) and the others (Figure 7A and 7B), whereas at 40 PSU, enzyme activity was seen to increase significantly in the CFH of mussels kept at 8.1 pH and 28°C (p<0.001) and 7.7 pH at both temperatures (p<0.05) with respect to the reference condition (8.1 pH and 22°C) (Figure 7C).

Significant effects of pH (p<0.001) and temperature (p<0.05) on the CFH protein levels were observed in clams kept at 28 PSU. Only temperature demonstrated a significant effect (p<0.05) on protein levels in mussels kept at 34 PSU, whereas pH and the pH/temperature interaction significantly affected protein concentrations at 40 PSU (p<0.001). Pair-wise comparisons indicated that at 28 PSU the CFH total protein concentration of mussels kept at 8.1 pH and 22°C was significantly higher with respect to animals kept at 7.7 pH and 22°C (p<0.01), and those from 7.4 pH and 22°C (p<0.001) and 28°C (p<0.01) (Table 3). No significant differences in the CFH total protein concentrations were recorded at 34 PSU. At a salinity of 40 PSU, total protein levels of mussels kept at 8.1 pH and 28°C and at 7.7 pH and 22°C significantly decreased (p<0.001 and p<0.01, respectively) compared with animals kept at 8.1 pH and 22°C (Table 3).

**Table 3:** Total protein concentrations (mg ml^−1^) in haemolymph from *M. galloprovincialis*.

*M. galloprovincialis*			
experimentalconditions	28 PSU	34 PSU	40 PSU
**8.1 pH, 22°C**	1.47±0.14	1.22±0.20	1.22±0.11
**8.1 pH, 28°C**	1.60±0.20	1.21±0.24	0.60±0.13***
**7.7 pH, 22°C**	1.19±0.17**	1.17±0.16	0.59±0.06**
**7.7 pH, 28°C**	1.29±0.18	1.36±0.24	1.21±0.17
**7.4 pH, 22°C**	1.15±0.11***	1.24±0.19	1.38±0.12
**7.4 pH, 28°C**	1.19±0.17**	1.35±0.11	1.28±0.15

Asterisks denote significant differences with respect to reference values (8.1 pH and 22°C temperature):

** p<0.01,

*** p<0.001; n. a.: not available. Results are means ± SE.

## Discussion

In bivalve molluscs, circulating haemocytes are involved in important functions, such as immune defence [20], [21], [26], [27] and shell deposition [28]. At an immunological level, non-self materials stimulate cell-mediated immune responses, which mainly include haemocytosis (increases in the circulating haemocyte number), phagocytosis or encapsulation of foreign particles (depending on their size), and the production of lysosomal hydrolytic enzymes (lysozyme in particular). Numerous studies have demonstrated that both biotic and abiotic factors can alter haemocyte functionality in bivalve molluscs, thus reducing their immunosurveillance.

The combined effects of pH and temperature on immune parameters of two bivalve species were evaluated in this study for the first time. The results obtained demonstrated that the experimental conditions tested altered significantly the cell parameters measured in *C. gallina* and *M. galloprovincialis*. Among the immunomarkers considered, THC varied markedly among experimental conditions. In bivalves, increases in the THC values are generally considered as a consequence of proliferation or movement of cells from tissues into haemolymph, whereas decreases are likely due to cell lysis or increased movement of cells from haemolymph to tissues [29]. Interestingly, in this study, a different pattern of variation in THC was generally observed between the two bivalve species. Using the reference condition of 8.1 pH and 22°C, exposure of *C. gallina* to 7.7 and 7.4 pH and 28°C significantly decreased the THC values at salinities of both 28 and 40 PSU. Conversely, the THC values generally increased in mussels kept at 28 PSU and 28°C and decreased at 40 PSU and low pH. Surprisingly, at a salinity of 34 PSU, the THC variation pattern was different between the two species, with THC generally increasing in clams and decreasing in mussels kept at low pH and high temperature values. These results indicated a modulation mechanism of circulating haemocyte number different between *C. gallina* and *M. galloprovincialis*, the former being more affected by stress conditions applied at 28 and 40 PSU, the latter being more affected at 34 PSU. Presumably, the decreased THC values recorded in *C. gallina* at the two extreme salinities were a consequence of an increased mobilisation of haemocytes from haemolymph into tissues. Similarly, a recent study demonstrated that exposure of the echinoderm *Asterias rubens* to 7.7 pH for one week reduced the total coelomocyte count significantly with respect to controls (8.1 pH) [30]. Conversely, the results of the present study are clearly in contrast with those reported by Bibby *et al*. [22] for *Mytilus edulis*, in which THC did not change significantly after 32 days of exposure at low pH values (up to 6.5) with respect to animals kept at 7.9 pH (reference value). The “time” factor significantly affected only the differential cell counts in *M. edulis*, with the percentage of circulating eosinophilic cells increasing markedly after 16 and 32 days [22]. However, it is important to highlight certain differences between this study and that of Bibby *et al*. [22]: i) the duration of the experiments; ii) the fact that no differential cell counts were performed in our study; and iii) the combined effects of changing environmental parameters were not tested by Bibby *et al*. [22]. Indeed, in Bibby *et al.*'s [22] study, the effects of acidification were only tested at 17°C and 34 PSU. Based on the results obtained in this study, we postulate that, at least in the case of THC, reduced pH and increased temperatures represent a stronger stress condition for clams than for mussels.

Numerous studies have demonstrated that the endocytotic activity (phagocytosis, in particular) of bivalve haemocytes can be affected by both exposure to xenobiotics and changes in environmental parameters [31]. In this study, the effects of pH and temperature on the NR uptake by haemocytes were assessed. This assay is faster than the phagocytosis assay, but equally responsive [32]. NR dye has been used extensively in *in vitro* assays to evaluate the effects of stressors on lysosomal membrane stability in bivalve haemocytes [33]–[36]. Uptake of this cationic dye by viable haemocytes can occur by pinocytosis or passive diffusion across cell membranes, whereas non-viable cells do not take up the dye [37]. Therefore, differences in the degree of dye uptake may reflect damage to cell membranes (including lysosomal membranes) and/or weakening of haemocyte pinocytotic capability. Our results demonstrate that exposure of *C. gallina* to reduced pH and increased temperature resulted in a different pattern of variation in haemocyte NR uptake between clams kept at 28 PSU and those at 40 PSU. Indeed, NR uptake increased at 28 PSU, but decreased at 40 PSU. A stimulation of NR uptake can be hypothesised for clams kept at 28 PSU; at this salinity value, it is possible that the stress due to reduced pH and increased temperature induced haemocytes to become more active, whereas the same stressful conditions reduced the THC in clams. To support this hypothesis, it is important to highlight that our previous studies demonstrated that exposure for 7 days of *C. gallina* to both hyposalinity (28 PSU) and hypersalinity (40 PSU) [38], as well as to high temperature (30°C) [39], significantly reduced phagocytic activity in clams. These results suggest that the variation of a single environmental parameter can induce a different cell response in contrast to those obtained when a combination of environmental factors is changed. Interestingly, in this study, clams kept at 34 PSU displayed a variation pattern in the NR uptake similar to that of bivalves maintained at 28 PSU. Therefore, it can be assumed that the combination of reduced pH and increased temperature is a more stressful condition for haemocytes at high salinity levels than at low and natural salinities. The effects of the experimental conditions on NR uptake were more obvious at salinities of 34 and 40 PSU for *M. galloprovincialis*, although the variation patterns were different. Indeed, NR uptake decreased in mussels kept at low pH and high temperatures at 34 PSU, whereas non-linear changes were observed at 40 PSU. In the same manner, phagocytosis (another endocytotic process) decreased significantly after 16 and 32 days of exposure of *M. edulis* to low pH values and 34 PSU [22]. Similarly, exposure for 7 days of *A. rubens* to 7.7 pH (12°C, 32 PSU) significantly reduced phagocytic activity of coelomocytes with respect to the controls (8.1 pH) [30].

Release of lysosomal hydrolytic enzymes is an important component in the extracellular killing of microorganisms by bivalve hemocytes [40]. Lysozyme is one of the most important bacteriolytic agents acting against several species of Gram-positive and Gram-negative bacteria; it is synthesised in bivalve haemocytes and subsequently secreted into haemolymph during phagocytosis [41]. In this study, lysozyme activity was measured in cell-free haemolymph to assess possible negative consequences of pH and temperature on enzyme release by haemocytes. The results obtained are difficult to explain, for two reasons: the absence of literature regarding the combined effects of pH and temperature on bivalve lysozyme activity and the non-linear responses of lysozyme in the bivalve species used in this study. In any case, exposure of *C. gallina* to reduced pH significantly lowered lysozyme activity in clams kept at 28 PSU, whereas pH/temperature interaction influenced enzyme activity at 28 and 34 PSU. Interestingly, a similar pattern of variation in lysozyme activity was observed between clams kept at 28 PSU and those maintained at 34 PSU. Conversely, the highest salinity tested in this study seemed to mitigate the effects of pH and temperature on clam lysozyme activity. In *M. galloprovincialis*, pH and temperature influenced haemolymph lysozyme activity in mussels kept at 28 PSU, with reduced pH generally decreasing enzyme activity and high temperature increasing enzyme activity. At 40 PSU, the pH and the pH/temperature interaction influenced lysozyme activity in mussel haemolymph, and enzyme activity was generally higher in stressed mussels than in the controls. These results suggest that there was a different response between *C. gallina* and *M. galloprovincialis* even when the animals were exposed to the same stress conditions. This can be due partially to different optimum values for lysozyme activity expression of bivalves under changing environmental parameters. In summary, it can be assumed that increased lysozyme secretion from haemocytes into haemolymph from both *C. gallina* and *M. galloprovincialis* likely occurred as a consequence of reduced cell membrane stability. However, attempts by the two bivalves to increase their respective immunosurveillance statuses at the peripheral level cannot be excluded. Conversely, animals under stress conditions may seek to reduce their energy expenditure through decreased lysozyme secretion.

Measurement of haemolymph total protein concentration is considered a useful biomarker to evaluate the health status of bivalves [42]–[44]. In this study, pH, temperature and their interaction significantly affected protein levels in haemolymph from *C. gallina* kept at 28 PSU. In particular, the lowest pH value tested significantly reduced haemolymph protein concentrations in clams. At natural salinity values, pH and pH/temperature interaction significantly influenced the protein concentration in clams; their levels increased at 7.7 pH and remained unchanged at 7.4 pH. In *M. galloprovincialis*, decreased protein levels were found in the haemolymph of mussels kept at reduced pH and increased temperature at the salinity of 28 PSU, whereas protein concentrations increased at the same pH and temperature conditions at salinities of 34 and 40 PSU. Reductions in the haemolymph protein concentration observed in the present study could be related to modifications of the metabolic activities in stressed bivalves, which reduced rates of protein turnover to lower energy expenditure. Indeed, metabolic depression is generally considered to be an adaptation strategy by animals to survive hypercapnia [45]. In this context, it has been demonstrated that hypercapnia interferes with protein turnover, resulting in lowered protein synthesis in aquatic organisms [46]–[50]. Conversely, increased protein levels recorded in *C. gallina* and *M. galloprovincialis* may be due to changes in haemolymph components; bivalve haemolymph (also known as serum or plasma) contains various proteins including soluble lectins, lysosomal enzymes, and various antimicrobial peptides [51]–[53]. Therefore, the activation of a variety of enzymes and/or proteins released from haemocytes into the haemolymph likely occurred in both clams and mussels, as reported for stressed oysters [54]. Further studies are needed to better understand protein expression profiles in haemolymph from bivalve molluscs under changing environmental parameters.

In conclusion, although it is not clear how pH and temperature affect haemocyte responses in bivalves, the results obtained in this study indicate that the experimental conditions tested can induce marked alterations in the immune parameters of *C. gallina* and *M. galloprovincialis*. Both reduced pH and high temperatures, which are predicted to occur in the coming years, affected the functional responses of haemocytes greatly, at times reducing the defence capacities of the stressed bivalves. This compromised state could lead to the increased susceptibility of animals to diseases. In any case, it is important to highlight that bivalves were exposed for a short time (7 days) to the combination of differing values of pH, temperature and salinity. The question is whether a long-term exposure may affect immune parameters of bivalves at the same extent. On the one hand, it can be hypothesised that the consequences could be more serious after long-term exposure of bivalves to the stressful conditions applied in this study. On the other hand, a great capability of animals to modulate their cellular, biochemical and physiological parameters in order to cope with stress conditions cannot be excluded. Overall, despite results obtained and the expectation that climate changes may affect the immunesurveillance status of animals, the present study does not provide a definitive prediction of how climate changes may affect the health and performance of bivalves. Further studies are needed to clarify better potential consequences of GCC on living organisms.

Considering that invertebrates represent approximately 95% of living species and play important roles in all ecosystems (aquatic, in particular), knowledge of the mechanisms involved in the immune modulation in animals will be increasingly important to develop management and conservation programs in future GCC scenarios. Further studies aimed at determining the effects (mainly the combined effects) of changing environmental parameters upon haemocytes of different invertebrate species are encouraged.
